# The Practicing Workshop: A Development Project

**DOI:** 10.3389/fpsyg.2019.02695

**Published:** 2019-12-05

**Authors:** Guro Gravem Johansen, Siw Graabræk Nielsen

**Affiliations:** Department of Music Education and Music Therapy, CEMPE, Norwegian Academy of Music, Oslo, Norway

**Keywords:** peer learning, instrumental practicing, practice and genre, deep learning, collaborative learning

## Abstract

In music academies and conservatoires, the culture of teaching and learning seems to nurture individuality and hierarchic structures at the cost of collaboration and sense of community. This could indicate a privatized conception of teaching and learning musical skills. As current research suggest that students’ learning may benefit from collaborative learning with their peers, the present article addresses music performance students’ understanding of the learning potential from participating in a specific social learning context. The study investigated students’ perceptions of outcomes from participating in a practicing workshop in a music academy, on both an individual and social level. The workshop aimed at helping music performance students develop their understanding of instrumental practicing. The course allowed for sharing ideas and experiences, and for collaborative exploration, discussion, and reflection. We conducted qualitative interviews with five volunteering students that had followed the workshop. Although identifying direct outcomes of the workshop in general proved to be difficult, we found that the students reported positive learning experiences from planning, implementing, and presenting their individual development projects, and that their awareness of variations in the group strengthened their confidence in how they tailored their own individual practicing. However, they also described more mixed outcomes in relation to learning about practicing directly from other students. The study also revealed that just providing a group forum for students does not in itself lead to positive learning experiences. Implications of the study are discussed.

## Introduction and Background

In previous research, the “conservatoire model” has been characterized as a cultural system that nurtures hierarchic structures and individuality at the cost of collaboration and sense of community (Kingsbury, 2001; see also Nielsen et al., 2018 for a brief review). Further, the belief that one-to-one studio lessons prepare for the student’s individual practicing sessions indicates a privatized conception of teaching and learning musical skills (Davidson and Jordan, 2007), where students are expected to take responsibility of their development on their own. In an ethnographic case study of a British conservatoire, Perkins (2013) found four distinct features characterizing the learning cultures in the institution. One of these was that a high degree of performing specialism, and “learning how to be an excellent performer” (Perkins, 2013, p. 203) was a central value in a physical environment that privileged one-to-one teaching and individual practicing. Further, students were concerned with building social networks. This concern was mainly focused on knowing important gatekeepers in work life (Nerland, 2004), rather than social engagement with peers for the sake of learning. Third, Perkins’ (2013) study confirmed Kingsbury’s (2001) claim that conservatoires are hierarchical institutions. Seen in relation to the fourth feature, the learning culture of vocational position taking, Perkins suggests that this conservatoire model may “[run] counter with the risk-taking and vulnerability central to learning” (Perkins, 2013, p. 209). Juuti and Littleton (2012) studied musicians’ transitions from the conservatoire to a professional work life. They describe how musicians were able to reconstruct and strengthen their learning processes and identity as musicians by acquiring a higher degree of agency and growth in confidence *after* they had finished their education. Poor conditions for risk-taking in a hierarchical and competitive environment, as described by Perkins (2013), may contribute in explaining the experienced lack of agency found in the study by Juuti and Littleton (2012). Thus, such educational cultures may be hindering learning for students during their time at the conservatoire.

In contrast to such learning cultures, current research indicates that students’ collaborative work with peers can benefit their learning (Hanken, 2016). In a survey that covered attitudes and perceptions about practicing among students in a Norwegian conservatoire, Nielsen et al. (2018) found that students reported an interest in using peers as learning sources, and that they thought it could benefit their development. However, their actual engagement in peer learning was lower than their interest and perceptions of benefit implied.

The concept of peer learning points to when members of a group of relatively equal status, such as students, “engage in mutual interactions with other students in order to learn” (Nielsen et al., 2018, p. 2). In music education literature, the view is commonly held that peer learning may be particularly suitable to provide a context for collaborative learning (Gaunt and Westerlund, 2013; Hanken, 2016). Collaborative learning means that the subjects in a group have a shared understanding of goals and a mutual engagement in problem solving (Gaunt and Westerlund, 2013, p. 4). However, as Nielsen et al. (2018) point out, a social peer context does not in itself guarantee that collaborative learning is happening. The development of a shared understanding and mutual engagement necessary for collaborative learning may be inhibited when the social environment is experienced as hostile or competitive, or when participants lack identification with each other (Bandura, 1997). Lebler (2007) describes an educational practice successful in nurturing peer learning in a popular music program, that in contrast to the previously described conservatoire cultures developed a “master-less studio” (p. 205). Instead of having the traditional instrumental one-to-one lessons, the program was organized as a collaborative peer-learning environment, where teachers had a less active role. A high degree of student autonomy was emphasized, facilitated through self-assessment by students, students giving feedback to each other, as well as teachers’ “genuine recognition of the students’ expertise” (p. 218). The horizontal feedback mechanism between peers nurtured experiences of safe failure and independence in learning. Lebler’s description of how students took social responsibility for their own processes shows how a peer-oriented, “master-less” approach may foster the student agency that is important for learning.

The comparison between the peer-oriented popular music studio and the hierarchical and competitive conservatoire cultures mainly coming out of Western classical music traditions indicates that learning cultures are dependent on genre. Previous studies have suggested that practicing practices are different according to genre, where classical conservatoire cultures seem to be more individually oriented and characterized by teacher-student relationships, while jazz and pop cultures as more socially oriented and characterized by peer learning (Creech et al., 2008; de Bezenac and Swindells, 2009; Sandgren, 2009; Johansen, 2016; Nielsen et al., 2018). However, we wish to warn against a simple classification of practicing cultures based on genre, as this may lead to confirmation bias and make us overlook variation as well as the potential of peer learning regardless of genres.

### Centre for Excellence in Music Performance Education’s Developmental Projects on Practicing Before and Now

Since 2013, NMH has hosted the Centre for Excellence in Music Performance Education (CEMPE). The center’s main objective is to initiate and conduct both innovative, exploratory teaching projects as well as research and development projects involving both students and teachers, to improve the quality of music performance education. Throughout the last 25 years, NMH’s research on practicing has been at the international research front, has been published internationally, and is widely cited (Jørgensen, 2009). This body of research includes studies on students’ management of practice time and their planning of practicing (Jørgensen, 1996, 2000); students’ learning strategies and motivation in practicing – characteristics of their self-regulated learning (Nielsen, 1999, 2001, 2004, 2008); jazz students’ practicing of improvisation (Johansen, 2016, 2018), and students’ preparations for performance as integrated in their practicing (Hatfield, 2017).

NMH’s research on instrumental and vocal practicing has constituted an important knowledge base in designing CEMPE’s practicing projects, and CEMPE (2019) now especially calls for projects with a focus on an expanded view on relevant learning resources for students’ practice activities in addition to that of the teacher (such as peers and new technology) as well as the importance of student-led developmental work (2019, p. 5).

## The Course: Aims, Content, Workshop Method

Within this context, CEMPE currently runs an elective course for students, starting August 2017, with the title *Development workshop in practising*, led by the two authors. It runs every year, and is open to all students across study program, year, genre, and instrument. The aim is to help students develop their understanding of instrumental practicing, by means of creating a collaborative forum for peer learning. The course is organized as a series of group workshops where students are provided input from a variety of angles and fields. This includes acquiring knowledge about different goals, approaches, techniques, and strategies from various genre domains and instrument groups, exploring transfer value from other domains such as sports and theater/acting, and, thus, helping the students improve their own practicing practices. The workshop format allows for sharing ideas and experiences, and for collaborative exploration, discussion, and reflection.

In addition to lectures held by us, we invited external and internal guest lecturers who are experts within different fields. Some of the topics that have been addressed are psychological skills and goal-setting; techniques for high achievement from sport science; practicing in contemporary music and folk music; mental “tagging” as a memorization strategy; health physiology for musicians; motivation, self-determination, and self-efficacy; practicing improvisation through exploration, self-regulation, and strategic practice; deliberate practice; and the relationship between identity development and high-level achievement from the perspective of acting.

Students from both Bachelor’s and Master’s programs elected the course, and they represented a genre background variety, covering jazz/pop, classical and contemporary music. As part of the course requirements, the students had to conduct a small-scale individual development project related to their own practicing. We encouraged them to articulate a problem that they felt was specifically relevant to their own practicing, and which was possible to investigate within the time and resource limits given. At the end of the course, they could choose between presenting the result of the project either as a written essay, or a video essay that might include practical demonstrations.

## The Study

In parallel with facilitating the course, we have run a development project within CEMPE with the course as the research case, and voluntary students as participants. The study aims at understanding students’ perceptions of outcomes of the elective course individually and as a result of social (peer) learning, and thus, the learning potential from participating in collaborative social contexts. The study was approved by the Norwegian Data Protection Official for Research.

In general, identifying learning outcomes in practicing is difficult, given that the development of instrumental skills, learning strategies and attitudes toward performance take time. Subjects are not always aware of slow and long-term changes at a given time. We are therefore approaching the purpose of the study by looking at how students reflect on practicing in general when encouraged to do so, within the context of the completed course.

Research question:

How do students reflect on practising in relation to individual challenges and goals, within the social context of a practice focused, collaboratively oriented course format?

### Participants

The participants of the study were recruited from two groups of students over a 2-year period. For each year, we asked the students to volunteer for an interview, by using informed consent. The students who signed up for the study filled out a consent form that stated the purpose of the study, their privacy, and the confidentiality of the information gathered. The first year, the initial design involved a focus group interview. The student group consisted of seven students, where the majority played classical music and one of them was a pop/rock student. Only two of the students announced their interest, one classical flutist and a classical pianist. The low number led to a change in the design. Firstly, a focus group interview could be experienced as intimidating with only two students, so we decided to carry out individual interviews instead. Secondly, with only two interviews we found the material too barren to provide any substantial results, which led to the decision to prolong the duration of the study to be able to recruit more students from the course the next study year. For the sake of methodological consistency, we decided to continue with the individual interview design. The second year, the course had six students. All of these were jazz students, except one, who played classical music. In this group, three of the jazz students signed up, on vocals, guitar, and bass, respectively. This gave us a total number of five student interviews.

The number of participants was lower than we had hoped for. Firstly, a higher number had potentially enabled us to carry out the initial design of focus group interviews. That way, the interview method itself would have reflected our interest in the social nature of reflection and learning. But even with individual interviews, a higher number of participants would have the potential of providing a larger variation in the data. The relatively low number thus represents a limitation of the study. We have no grounds to establish whether the variation we have been able to cover in students’ experiences is representative, and thus, the findings should not be generalized.

Although the genre specialization among the participants did not cover the range of genres that are present among students at NMH (which includes folk music, rock, pop, experimental, free improvisation), two main genre domains (classical music and jazz) are represented. Further, the instrument variation was high. The low total number also had the advantage of enabling us to treat the participants as individual cases and looking in detail at each participant’s experiences and perspectives. Hence, in the analytical stage, we have applied a cross-case design (Stake, 1995).

### The Interviews

Conducting research with participants who are also the researchers’ students holds an ethical dilemma. Both the students and the two researchers held double roles toward each other, in asymmetrical power relations. Students may restrict their talk or actions toward teachers who are in the position to exert some degree of power, such as course assessment. Furthermore, as teachers in a course about practicing, it was possible that students perceived us as having certain standpoints and recommendations on the topic, despite intentions of a non-normative attitude on our behalf. These mutual constructions of each other’s roles potentially influenced the degree of openness and trust in the interview situation.

To limit a potential mix-up of roles, the interviews were carried out after the course and final assessment had finished for each of the years. Further, we made explicit both verbally and in the consent form that we separated between their contributions during the course and in the interview; and that in the interviews, there were no right or wrong answers.

As mentioned, we were interested both in how students perceived and articulated their learning outcomes of taking the course, and what practice-related topics emerged as a result of talking about outcomes; what do students bring up when they are encouraged to talk about practicing? The interview guide had three sections, with questions functioning as providing a broad direction, in accordance with the individual, thematic flexibility needed for semi-structured, qualitative interviews (Kvale and Brinkmann, 2009). In the first section, we wanted to focus on changes in goal-setting and practice behavior over the last study year, in addition to experiences of collaborative learning, using the following guiding questions:

1. What are your current goals for practicing?
2. Do you practice differently now compared to before?
3. How do you practice today? Has it changed? If yes, why?
4. What are your experiences of discussing practicing with others?

In the second section of the interview, we presented a list of all the themes covered by the course that actual year, and asked the students to pick out the ones they felt had particularly contributed to a general learning outcome in the course. This way, we wanted to trigger their memories and incite a more general reflection on their perceived outcomes. In the third section, we asked the students to talk about the perceived outcome of (1) conducting their individual development project and (2) hearing about and engaging in the other students’ projects (to the degree that they had). An important question is whether students’ reflections in the interview situation were results of having participated in the course, or whether they were incited by, and emerging in, the interview situation itself. We want to be careful not to attribute students’ reflections around their practicing in the interviews to be a direct effect of the course. Most likely, the results demonstrate a combination of having attended the course and being in the interview situation. The course provided a social arena for sharing and collaboratively reflecting on the issues raised. Since the interviews were carried out after having attended the course, students’ reflections show their thinking at the time of the interview. As was the case with the course, the interviews represented a situation where meaning was constructed through social interaction. Thus, participating in the course may be seen as a context where students had *practiced* talking about and reflecting on practicing through social interaction, an experience that may have enhanced their ability to verbalize their thoughts and perspectives in the interview.

### Analysis

The interviews were transcribed using an intelligent verbatim transcription done by a professional firm named *Totaltekst*. This firm is hired by the academy to do these kinds of tasks in accordance with the GPDR. The transcripts were checked by us. An important topic for each interview was the students’ individual course projects. Therefore, we have conducted analyses with a twofold purpose; firstly, a holistic interpretation of each individual case has been carried out, but with an instrumental purpose in order to help us understand the phenomenon of practicing and peer learning in practicing (Stake, 1995). Secondly, we have categorized the interviews with a cross-case thematic focus. In the categorization process, we have focused on individually distinct as well as overlapping themes, regarding *what* issues they talk about, and *how* they talk about them. We identify the participants by their instruments. The participants’ gender has been randomized using a shuffle algorithm (Coyne, 2019).

## The Five Cases

### Classical Flutist

Student 1 is a flutist in the Western classical music program. He initially described the past academic year as a “chaotic” one as he had played a lot of auditions and exams, but his main goal had been to become better in planning and preparing for these situations. Especially, he had found it beneficial to learn how to use mental practicing techniques such as goal-setting, visualization, and coping strategies in practicing situations in order to feel more comfortable and less self-critical in the performance situations. Getting to know how to use these techniques became his main focus area also in the practicing workshop, and as he pointed out: “Now I realise that putting away the instrument and practising visually also can be very useful. So, when I was on the plane to [name of city] yesterday, I thought about the music excerpts [for the audition] and how I wanted to play them, and it became a kind of practicing for me. Previously, I would not consider this as practice.” Now he also finds it more necessary to articulate specific goals concerning musical decisions. Earlier he often became distracted by technical challenges that occurred there and then instead of keeping the musical phrasing in focus. Even if structure, goal-setting, and a more deliberate focus are important to the flutist now, he also acknowledges the value of taking liberties in his practicing such as improvising on scales, to create variation, and keeping up his motivation. The flutist also points out how he struggles to avoid feeling guilty when not practicing, for example during holidays. Although his teachers encouraged him to take time off, he does feel a pressure to follow the routine of practicing every day, a pressure that is both self-imposed and coming from other students. Although he preferred to have structure in his practicing, he had experienced through participating in the practicing workshop that practicing can be manifold, and that it is “possible to do different things and that it still may work.” The flutist did not make any comments regarding discussing issues on individual practicing with peers.

### Classical Pianist

Student 2 is a pianist in the Western classical music program. He claimed that he had not changed his way of practicing much in the last 5 years; nor in the last year when he participated in the practicing workshop. However, when he began his studies at the bachelor’s program 5 years ago, he began rehearsing a new piece by first listening to recordings of the piece made by acknowledged performers, and then, practicing the piece from the beginning and “trying to make it [the piece] sound something like that [the recorded interpretations].” Now he challenges himself to learn a new piece by first reflecting on and understanding the score and the music as if asking the score: “What are you telling me?” He also uses mental practicing in order to acquire an emotional relationship to the piece from the very beginning. Earlier he experienced a high degree of pressure and hurry in regard to learning new repertoire, and thus, never feeling sufficiently prepared when performing, which in turn had created a high risk of failure, and thus, a fear of failure in him. His experience was that “if rushed when learning a new piece, one runs the risk of rehearsing mistakes.” Thus, now he tried to take time when learning a new piece, such as practicing every movement slowly and simultaneously automatizing the movements while feeling a sense of constant positive mastery. Although he now articulates both short-term goals and long-term goals, the overriding goal of practicing for him is the process of practicing itself: “I play the piano to have the opportunity to practice. It’s amazing to play concerts, but that is not why I play.” When it comes to discussing issues on individual practicing with peers, he would have appreciated to do that more often as he found it valuable and important to “get new inputs or just be reminded of something you may have forgotten.” In addition, he would have found it very valuable if his piano teachers also had shared and discussed their learning experiences with him to a greater extent, or that he could have been encouraged to ask them to reflect on their own practicing of specific pieces. However, being a student in the practicing workshop, he more often had felt that he had positioned himself as the one giving advice to the others instead of benefitting from hearing about the others’ experiences into his own practicing.

### Jazz Singer

Student 3 is a singer in the jazz/improvised music program. In the opening sessions of the practicing workshop, she stated that she did not practice at all because she did not know how. The reasons behind this statement were several. One reason was that she felt she had little experience with getting help to find practicing strategies – that is, what kind of musical material, exercises etc. she should practice to develop as jazz singer. Another reason was that she did not think that just playing with her voice – that is, exploring different techniques or ways of singing just for fun, could be defined as practice. At the end of the semester, she experienced that both developing as a singer and making songs, both were important goals for practicing sessions. Especially, she found that developing *ownership to the musical material* and *ownership in ways of practicing* proved to be key motivations in developing routines for practicing. Now playing and fooling around with the voice or on the piano had become a deliberate strategy for exploring who she wants to be as a musician. In addition, the singer now defined reading scores as an important part of practicing, to prepare for gigs with ensembles such as big bands or vocal ensembles. Such gigs often require short practicing time with the ensembles before concerts. The singer points out that earlier, she did not practice enough before such gigs as she had thought that using the score and her voice alone was insufficient to rehearse her parts in written music: “I can’t do anything unless I have some recordings to sing along with.” The singer had not experienced peer learning in practicing earlier – that is, thinking that she did not practice, she had nothing to share with others on this matter: “When I do not have any experience with practising, I do not seek out people to talk about practising.” However, being a student in the practicing workshop she experienced that she could observe what others did and how they reflected on their own practicing. “This proved to be very interesting in developing my own routines.” The singer also pointed out how talking about how students with other instruments and/or genres than her own has been fruitful, in that she has realized how different approaches to practicing may prove valuable for different people: “I need to find the approach that works for me, and if it works for me, then it is the best or most efficient method in the world.”

### Jazz Bassist

Student 4 is a double bass player in jazz/improvised music. She stated initially that one of her goals was to improve her practicing of intonation. Although she acknowledged the need to develop a solid intonation in order to become a better double bass player, practicing technique felt boring, which was her major challenge. Consequently, she wanted to design a peer-learning environment to motivate herself for this kind of practicing throughout the semester. The double bass player had previously used what she called “buddy-practicing” when practicing technique. Then, she practiced together with another double bass player, and this had worked well in order to get the task done and to keep concentration during task performance. Another goal was to integrate approaches from how to improvise solos on other instruments into her own improvisation on the double bass. The challenge was to be able to visualize and transfer approaches to improvising onto the double bass from, for example, the piano, where “the left hand plays chords offbeat while the right hand plays melody or the other way around.” Lastly, her goal was to play and do gigs in a lot of different bands and projects in order become more established as bass player in the professional jazz community. However, she realized that this goal could lead to less time for individual practice and preparations for the soon upcoming exams. The double bass player had also experienced peer learning in practicing beyond the “buddy practicing” situations. She particularly highlighted earlier experiences in playing in a free jazz trio. In trio rehearsals, she recalls how the musicians mainly used the rehearsal time to discuss and reflect on what was to be played and on the need to trust their own musical ideas and each other, rather than on actually practicing playing the music: “It was a new and very interesting experience, especially in the context of playing free jazz.” However, when it comes to discussing issues from individual practicing with peers, the practicing workshop was a new experience. The double bass player found it interesting “to notice what kind of situations other [students] are in when practising, and that it also was a bit validating and nice to see how they also used completely different approaches.”

### Jazz Guitarist

Student 5 is a guitar player in jazz/improvised music. He described how he often sets specific goals for his practicing and works with different details in periods over 2–3 weeks. However, he also stated the following: “I have the same goals as I have always had. Those things you are never finished with.” At the end of the semester, this was still the case:

There is so much to be improved all the time, but it is rare that new things come up, in a way. You can't be the best at everything either, so you have to prioritise at some point.

The guitar player had quite early on begun to make notes on what he missed in his own playing, like expanding the dynamic range (inspired by John Scofield), how to better “blend in” in an ensemble, keeping time and beat better, and how to phrase more “legato-like” as well as keeping several melodies going at the same time in his solos. Since exams are coming up at the time of the interview, he needs to become more efficient in her practicing. Thus, he has to set very specific goals for each practicing session. A useful strategy in this regard is to make recordings of himself, and to listen through his playing repeatedly. In all, the student claims to be very self-driven in his practicing and is highly motivated by this fact. In discussing practicing in the workshop group, he found it beneficial to get to know the other students’ different approaches to practicing, but from hearing about the others’ practicing, he became even more confident that he had established the approach that worked best for him. However, he was a bit surprised to find several similarities between his practicing and the approach of the classical pianist in the group as she also “goes down to a very micro level. Playing things over again. Since they [classical musicians] have written music, they can to a greater extent work like that.”

### Cross Case Analysis: Outcome of the Course and Changes in Practicing Behavior

As seen in the case presentations, the variation in practicing habits, attitudes, and problems related to practicing were great across the students. In this section, we will address some themes in a cross-case analysis that relates more specifically to their perceptions of the course and its outcomes. One of the main purposes with the course is that through learning about different approaches to practicing across individuals, genres, and instrument traditions, as well as from research, the students can develop their own practicing practices. Ultimately, this may be seen as perceived changes in their practicing habits after the course has ended.

The perceived outcome of the course appeared to depend on the degree to which they found a respective topic relevant to their own, individual needs. Simply hearing about practice-related topics in lectures that were not perceived as directly relevant did not result in major learning for the students, even if they found the topics interesting. Further, it may be the case that relevance to the students meant for something to have an immediate utility in relation to already identified problem areas for the individual. To contrast this attitude, it is possible to find relevance in themes and perspectives that are more distanced from a student’s existing practice, if experienced as inspirational and opening possibilities for exploring problem areas new to the students. However, the latter may involve a sense of risk taking and vulnerability. Thus, an individual “utility view” may be seen as confirming a culture of individualized specialism, where risk taking is inhibited (Perkins, 2013). Hence, these positions reveal that the shared understanding that characterizes collaborative learning (Gaunt and Westerlund, 2013) was limited. From one perspective, the lack of finding relevance in hearing about other students’ practicing may be seen as a lack of identification among peers, which in Bandura’s (1997) social learning theory is an important precondition for collaborative learning. However, as seen in the previous section, both S3, S4, and S5 found it more beneficial to hear about other students’ practicing than S1 and S2, but not necessarily because they found others’ approaches directly applicable to a higher degree. For example, S5 said that hearing about students with very different problems and attitudes made him even more confident that the habits he had established were the right ones for him. Hence, learning about the variation in the student group was beneficial in the sense that it validated individual differences. This validation may in fact enhance identification, through an acknowledgement of diversity.

In the interview, we asked the students about whether their practicing had changed during the school year. Predominantly, they expressed that practicing habits had not changed much during the last year, and not as a direct consequence of the course. As S5 put it: “I do much of the same. My goals now are very similar to before.” S2 said: “I haven’t changed much the last year. I can tell you more about changes in my practising over the last five years.” However, S4 said that she often considered many of things she had picked up during lectures when she practiced: “I don’t remember the details particularly well, but the different models we were presented to, they are sort of in the back of my head. I think about it when I go into the practice room.”

S1 said that in order for new techniques to actually influence his practicing, it was not enough to hear about them; he needed to test them out in practice – to practice them. In a similar vein, S2 pointed to how perception of relevance was connected to whether the topic in question was presented in a theoretical way, or whether it was experienced in practice. He said that the course had had “too much talk, and too little practical work.” In S2’s view, even content that is not directly useful to his instrument or genre is still interesting as long as it is practically oriented. He phrased it like this: “Understanding doesn’t bring results, other then perhaps making the work easier to do afterwards. But doing the work is much more important. You can’t read a book and then go and play the piano. You actually have to practise for real. If you’re going to dig a ditch, you have to pick up a shovel.”

As part of the course, we as teachers initiated sessions where we wanted the students to discuss each other’s projects, hoping that group engagement on their different projects would turn out beneficial to them. However, although S1 did enjoy it, he found engaging in such discussions difficult, because he did not always feel he had much to contribute with on other students’ problems. On the other hand, S2 did not experience learning a lot from discussions as he found himself more experienced than the others, as seen in the case description. Hence, S1 and S2 both expressed low learning outcomes from group engagement, but explained this experience by positioning themselves in different ends of a hierarchy; S1 felt too inexperienced to contribute, and S2 felt too experienced to benefit. These students’ inclination to position themselves in this way may be seen as an internalization of the hierarchical and specialist learning culture identified in some of the conservatoire studies (Kingsbury, 2001; Perkins, 2013). Thus, the degree of individual achievements or experience determines an individual’s status or “place” within a social group, and may hinder peer identification with students with a different status.

The opportunity to define an individual project as part of the course was highlighted as the main thing that they experienced as having the greatest outcome, and leading to the biggest changes. Then they could focus on what each felt they needed the most. S5 wanted to become better at creating strong melodies in his improvised guitar solos. He said that his chosen project did not represent a new problem to him, but that the task forced him to articulate it in a clearer way, as well as be concrete about his strategies to work with it. S3 had chosen to reflect on how an increased self-determination could impact her motivation to practice, and thus actually lead to increased practice. She said that learning about the connection between self-determination and motivation “was like a wake-up call” to her. When she started writing down her thoughts related to the topic, the change was almost existential to her.

## Conclusion and Discussion

### Summary of Findings

In this study, we aimed at investigating the students’ outcomes of participating in the practicing workshop on both an individual and social level, with the broader aim of understanding the learning potential from participating in social contexts.

As suggested from earlier research, “collaborative forums for instrumental practising within HME institutions can function as constructive and supportive arenas to enhance students learning and inner motivation” (Nielsen et al., 2018, p. 1). Summing up, our findings indicate that students did not perceive their practicing as having developed radically over the year the course lasted. They also expressed a varying degree of experienced learning outcome from the course. However, there was a difference between perceived outcomes from their individual development projects, which was relatively high, and the outcome they experienced from sharing and discussing issues related to practicing with each other, which was more mixed, depending on how individually relevant and useful students found various topics to be. Nevertheless, becoming aware of differences and variation in the group was experienced as beneficial, and previously we have suggested that processes where diversity is acknowledged can enhance group identification.

Some also found it difficult to engage in mutual discussions, for various reasons. As seen before, we have suggested that this may be due to internalized values resting in conservatoire learning cultures as identified in previous research, cultures that nurture individualization and hierarchical positioning. These factors may have inhibited a sense of identification, and thus also learning.

There may be several reasons for why identifying direct outcomes of the workshops seemed difficult in general. Partly, it is potentially difficult to be aware of and articulate potential changes even if they happen. Partly, the difficulty in addressing direct outcomes may be seen as an indication of how changes in practicing behavior may take longer time than just 1 year, given the high level and presumably long-time habits conservatoire students have established before they take such a course.

Regarding the individual projects, all of the students highlighted how their projects had led to development and learning. While this may be seen as self-evident, it is well worth looking at why this was the case, and we wish to suggest several factors. The students were allowed an unlimited scope in regard to what they considered relevant to their practicing, and in how they articulated their project problem or focus. This led to a wide range of topics, where some were almost existential and related to identity, whereas other projects were quite specific. This way, ownership, motivation, and thus engagement for carrying out the project were presumably high. Furthermore, the requirement for the project was to carry out some form of action in practice, to document the process and present it to the teachers and the group, but we did not demand that the actions undertaken had to lead to observable results. Thus, we expected that the students experienced a high degree of freedom and allowance for potential negative or non-existing results, with the potential “relief” of not being assessed for the degree of actual development as performers or change in habits. This responds to both Lebler’s (2007) and Perkins’ (2013) call for learning environments with a perceived low risk of failure.

To what degree the individual projects had an impact on students’ collaborative learning should be discussed. As we saw, some students found topics that were remotely related to their own musicianship less interesting, which again underscores the importance of being able to determine one’s own foci such as through the individual projects. Nevertheless, the social dimension of carrying out the individual project is also important to highlight. Their project work happened within a social framework of peers and teachers, where this framework required that all had to share their own thoughts and ideas for projects. Even if the projects did not necessarily represent new issues to the students, there was a potential for learning resting in the need to articulate the problem, strategies, and results in a clearer way in order to communicate them to others. Thus, the requirement to communicate may have become formative of one’s own understanding of the problem at hand. Therefore, we argue that doing individual projects within the forum of a group can lead to an indirect social and collaborative form of learning.

As for the second dimension, the perceived outcome of discussing practicing, student’s responses were varied. Even if hearing about other students’ practicing behavior did not feel directly relevant for one’s own practice, this feature did not directly affect how they experienced the learning outcome to be. It is noteworthy that the participants from the first year’s interviews experienced a lower outcome in general than the participants from the second year, who expressed a more positive attitude regarding outcome.

Although the total number of participants is too low to draw firm conclusions, some differences between the two student groups are evident. Firstly, both S1 and S2 were classical musicians, and S3, S4, and S5 were all jazz musicians. It may be the case that jazz students in general are accustomed to discussing practicing with each other than classical, and that they therefore are more used to eliciting information from others they can use for their own benefit. This is suggested by previous research (Creech et al., 2008; Nielsen et al., 2018), although Johansen’s (2014) study on practicing among jazz students indicates that there is no consistent pattern of peer learning among these.

Secondly, the two groups had followed the course in two slightly different versions. As shown from the interviews with S1 and S2, they both called for a more practically oriented, less talk-oriented design of the lessons. Therefore, in the second year, we specifically ensured more student-active lessons, where we had them to try out and explore various issues and problems directly on their instruments. In our observations, this led to more discussions and engagement.

It is possible that playing and singing exercises together demanded a willingness to be vulnerable and take risks in front of each other, factors that are crucial to learning (Perkins, 2013). The intimate and embodied engagement with a problem that playing requires may function as a bridge between students’ different experiences and perspectives. This activity may thus foster a shared understanding (Gaunt and Westerlund, 2013) and a stronger sense of identification among students (Bandura, 1997). Further, when students themselves had been active, their subsequent reflections were possibly more related to each other’s contributions in the class, and less to the lecturing of teachers. It is fair to assume that this aspect led to a deeper engagement and interaction among the peers.

This aspect underscores the importance of doing in learning, and that constructing a social and collaborative setting is not enough if the teaching and learning activity is dominated by talk at the cost of practical experience. Practical experience combined with critical reflection may provide opportunities for deep learning in relation to particular issues (Entwistle, 2000; Leung and Kember, 2003). This phenomenon was underscored by students’ positive learning experiences of their individual projects.

## Implications for Higher Music Education

Instrumental practicing is often considered a core activity in higher music education (HME), and therefore we suggest that educational development and change in this area may have an important impact on the quality of HME in a broader perspective. In line with previous research, our study confirms the potential for learning in collaborative forums, if it is rigged in a way that fosters mutual identification and shared understanding, while at the same time allowing for individual differences. Further, creating collaborative forums centering on this core activity may have the potential to challenge cultural values in the conservatoire that may be inhibiting to learning, such as the tendency for hierarchical positioning, a competitive and individualistic environment of specialism, and when failure is experienced as high risk (e.g. Perkins, 2013).

Based on the present study, we wish to suggest certain features of how such forums can be facilitated in order to foster collaborative learning. First of all, if students are not used to talking about practicing and reflecting on it collaboratively, then this process may require practicing in its own right. Second, allowing students to address problems of their own choices in individual development projects along with the requirement to invest time and effort in this is a way of ensuring a sense of relevance and ownership. Further, we recommend that giving space to individual issues is accompanied by a non-normative attitude toward diversity in regard to practicing. Our study indicates that acknowledgement of differences in processes of sharing is important to enhance mutual identification in a group, and also that the effort required to communicate one’s own perspectives to others with different perspectives is an important dimension of collaborative learning.

Another feature that has proved to be important for students’ experienced outcome is taking a practical approach where playing activity is foregrounded. Thus, this study reminds us that learning practicing is both about the individual student’s “permanence of having” knowledge on practicing, but also about the “constant flux of doing” (Sfard, 1998, p. 6), understood as doing practicing and making considerations on practicing in a learning community where students and facilitators may affect and inform each other.

## Data Availability Statement

The datasets generated for this study are available on request to the corresponding author.

## Ethics Statement

The studies involving human participants were reviewed and approved by the Norwegian Social Science Data Services. The patients/participants provided their written informed consent to participate in this study.

## Author Contributions

GJ and SN have designed and conducted the case study, conducted the analysis, and written the article. The study was funded by CEMPE.

### Conflict of Interest

The authors declare that the research was conducted in the absence of any commercial or financial relationships that could be construed as a potential conflict of interest.
